# The T790M resistance mutation in EGFR is only found in cfDNA from erlotinib-treated NSCLC patients that harbored an activating EGFR mutation before treatment

**DOI:** 10.1186/s12885-018-4108-0

**Published:** 2018-02-15

**Authors:** Christina Demuth, Anne Tranberg Madsen, Britta Weber, Lin Wu, Peter Meldgaard, Boe Sandahl Sorensen

**Affiliations:** 1 Department of Clinical Biochemistry, Aarhus University Hospital, Palle Juul-Jensens Boulevard 99, 8200 Aarhus N, Denmark; 2 Department of Oncology, Aarhus University Hospital, Norrebrogade 44 bld. 5, 8000 Aarhus C, Denmark; 3 Roche Molecular Solutions, Pleasanton, CA 94588 USA

**Keywords:** Carcinoma, Non-small cell lung, Receptor, Epidermal growth factor, Erlotinib, Drug resistance, T790 M

## Abstract

**Background:**

Lung cancer patients with an activating mutation in the EGFR (epidermal growth factor receptor) can develop resistance to erlotinib treatment, which is often mediated by the T790M resistance mutation in EGFR. The difficulties in obtaining biopsies at progression make it challenging to investigate the appearance of the T790M mutation at progression in large patient cohorts. We have used cell free DNA (cfDNA) from patients treated with erlotinib to investigate if the development of a T790M mutation coincides with the presence of an activating *EGFR* mutation in the pre-treatment blood sample.

**Methods:**

A cohort of 227 NSCLC (non-small cell lung cancer) adenocarcinoma patients was treated with erlotinib irrespective of *EGFR*-mutational status. Blood samples were drawn immediately before erlotinib treatment was initiated and again at progression. The cobas® EGFR Mutation Test v2 designed for cfDNA was used to identify 42 *EGFR* mutations.

**Results:**

Of the 227 NSCLC patients, blood samples were available from 144 patients both before erlotinib treatment and at progression (within 1 month before or after clinical progression). One hundred and twenty-eight of the 144 were wild-type *EGFR* before treatment, and we demonstrate that the T790M mutation was not present at progression in any of these. In contrast, in the 16 patients with an activating *EGFR* mutation in the pre-treatment blood sample six patients (38%) were identified with a T790M mutation in the progression blood sample.

**Conclusion:**

The T790M resistance mutation is only found in the cfDNA of erlotinib-treated NSCLC patients if they have an activating *EGFR* mutation before treatment.

**Electronic supplementary material:**

The online version of this article (10.1186/s12885-018-4108-0) contains supplementary material, which is available to authorized users.

## Background

A subgroup of NSCLC patients have activating mutations in the *EGFR* gene, primarily in exon 19 and 21 [1–3]. This group responds well to EGFR-directed tyrosine kinase inhibitors (TKIs), like erlotinib and gefitinib, and today these agents are part of standard care. Despite an initial response, all patients eventually acquire resistance to the treatment [4–6]. One defined mechanism causing resistance to TKI treatment is the development of the T790M mutation in exon 20 of the *EGFR* gene [7, 8]. Additionally, it has been shown that amplification of the *MET* (met proto-oncogene (hepatocyte growth factor receptor)) gene or increased expression of HGF (hepatocyte growth factor) can cause resistance (reviewed by Tartarone et al. [9]).

Apart from the NSCLC patients with activating *EGFR* mutations, it has been suggested that a subgroup of the *EGFR* wild-type patients also experience benefit from erlotinib treatment [10]. These patients ultimately develop resistance as well. Not much is known about the resistance mechanisms for this patient group. Generally, investigation of resistance mechanisms for both *EGFR* wild type and mutation-positive patients is complicated, since re-biopsies from patients with progression are not taken systematically.

In the present study, we have taken advantage of a new possibility of detecting the T790M resistance mutation in a blood sample [11]. We used the cobas® EGFR Mutation Test v2 designed for cell free DNA (cfDNA) to investigate the presence of the T790M mutation in the cfDNA from NSCLC patients treated with erlotinib.

## Methods

### Patients and blood samples

From a previously collected cohort, 227 NSCLC adenocarcinoma patients treated with erlotinib from October 2008 to December 2012 were selected based on the presence of both a biopsy and paired blood samples isolated during treatment [10]. The project was approved by the Central Denmark Region Commitees on Biomedical Research Ethics (M-20080012). All included patients gave written consent.

Blood samples were collected immediately before erlotinib treatment was initiated, and again when treatment was terminated due to progression as defined by RECIST (response evaluation criteria in solid tumors) version 1.1 criteria or by clinical deterioration defined by the treating physician. Patients without progression at first follow-up with CT-scan (3 months after therapy initiation) were considered responders [10].

Of these 227 patients, 144 patients had paired blood samples before erlotinib treatment and within 1 month before or after progression. A total of 16 of the 144 patients had an activating mutation in the *EGFR* gene in the pre-treatment plasma DNA sample, as well as in the tumor biopsy taken at diagnosis, whereas 128 were *EGFR* wild type. None of the patients had a T790 M mutation in their tumor before treatment. Patient characteristics can be seen in Table 1. Blood and biopsy sampling, and identification of *EGFR* mutations using the cobas® EGFR Mutation Test v2 (Roche Molecular Systems, Inc., CA, USA) have previously been described [11, 12]. The test contains positive and negative controls and all runs passed this quality check. CfDNA was isolated from 2 ml of plasma and eluted in 100 μL with the cobas DNA sample preparation kit (Roche).

**Table 1:** Patient characteristics

Variable	Value	
	Number	Percent
Age (years)		
Mean	65	
Range	34–87	
Gender		
Male	78	54
Female	66	46
Ethnicity		
Asian	1	1
Caucasian	143	99
Smoking history		
Current smoker	49	34
Former smoker	85	59
Never smoker	10	7
ECOG performance status^a^		
0	18	12
1	80	56
2	37	26
3	9	6
Tumor type		
Adenocarcinoma	126	88
Not otherwise specified (NOS)	9	6
Adenosquamous carcinoma	5	3
Large cell	3	2
Adenocarcinoma in situ	1	1
Tumor stage		
IIIA+IIIB	5	3
IV	139	97
Erlotinib therapy		
First-line	10	7
Second-line	115	80
Third-line	14	10
Fourth-line	5	3

^a^Eastern Cooperative Oncology Group scale to assess how the disease affects the daily living abilities of the patient

### Droplet digital PCR

Droplet digital PCR was performed on the QX200 droplet digital PCR system (BioRad). Five μL of the cfDNA isolated as described above was used for ddPCR and all samples were analyzed in triplicate. Validated kits for both the wild type and the T790M-mutated *EGFR* were purchased from BioRad (cat #10031250 and cat #10031247) and the assay was performed with the ddPCR Supermix For Probes Reagents (BioRad, cat #186–3025). Results were analyzed with the QuantaSoft software (Bio-Rad).

### Cell lines and reagents

Wild-type *EGFR* adenocarcinoma NSCLC cell lines Calu-3 (ATCC® HTB55™), H358 (ATCC® CRL-5807™), H1568 (ATCC® CRL-5876™), and H1666 (ATCC® CRL-5885™) were purchased at ATCC. Calu-3 was cultured in MEM media (Gibco, Lifetechnologies) and H358, H1568, and H1666 were cultured in RPMI-1640 media (Gibco, Lifetechnologies) according to the manufacturer’s instructions. Erlotinib-resistant cell lines were established by two methods; step-wise escalation of drug (ER), where the drug concentration was escalated from 10 nM to 5 μM, and high dose drug (HD), where the drug concentration was fixed at 5 μM. Cells were considered resistant after 4–6 months when they were able to grow in 5 μM erlotinib (Selleckchem). Cell viability studies were performed using CellTiter 96® AQueous Non-Radioactive Cell Proliferation Assay (Promega) following manufacturer’s recommendations.

### Statistics

The Fisher’s exact test was used to assess the hypothesis of no difference in risk of gaining the T790M mutation between patients with and without an activating *EGFR* mutation. All statistics were calculated in Stata 13. Data obtained from cell viability studies are presented in graphs produced in Prism 6.

## Results

One hundred and twenty-eight patients with wild-type *EGFR* in the pre-treatment blood sample were identified among the 144 included patients. None of these patients had developed the T790M resistance mutation in the blood sample taken at progression (Table 2). Among the 16 patients harboring an activating *EGFR* mutation in the pre-treatment blood sample, the T790M mutation was found in 6 (38%) of the blood samples taken at the time of progression. Table 2 shows the frequency of the T790M mutation in blood samples taken at the time of progression in patients with or without activating *EGFR* mutations in the pre-treatment blood sample. Statistical analysis demonstrated that the T790M mutation was preferentially associated with the presence of an activating *EGFR* mutation (*p* < 0.0001, Fisher’s exact test). Also, we investigated the distribution of the T790M mutation in patients with defined clinical response to treatment (see Table 3). Again, the T790M mutation was associated with an activating mutation in EGFR (*p* < 0.0001, Fisher’s exact test).

**Table 2:** The T790M mutation in progression blood samples

	Progression sample		
Pre-treatment sample	No T790M	T790M	Total
Wild-type *EGFR*	128	0	128
*EGFR* mutation	10	6	16
Total	138	6	144

**Table 3:** The T790M mutation in progression blood samples from patients with defined response

	Progression sample		
Pre-treatment sample	No T790 M	T790 M	Total
Wild-type *EGFR*	31	0	31
*EGFR* mutation	6	6	12
Total	37	6	43

To further substantiate these results, we performed droplet digital PCR (ddPCR) with DNA from the paired blood samples of the 16 patients with activating *EGFR* mutations to detect the T790M mutation. The T790M mutation was not present in any of the pre-treatment blood samples as seen with the Cobas assay. Complete concordance was also apparent between the results from the cobas® EGFR Mutation Test v2 and ddPCR in the progression blood samples (Table 4).

**Table 4:** Comparison of Cobas® and ddPCR for T790M detection in paired blood samples

	Cobas® EGFR Mutation Test v2		
ddPCR	No T790M	T790M	Total
No T790M	26	0	26
T790M	0	6	6
Total	26	6	32

To investigate whether *EGFR* wild-type NSCLC cell lines developed T790M-mediated resistance, we established two different in vitro cell line models of erlotinib resistance with four *EGFR* wild-type NSCLC cell lines. Erlotinib response of all parental and resistant cell lines can be seen in Additional file 1: Figure S1. None of the eight resulting erlotinib-resistant cell lines developed the T790M mutation, or any other secondary EGFR mutation, as a resistance mechanism to erlotinib when analyzed with the cobas® EGFR Mutation Test v2.

Our results demonstrate that the T790M mutation is only detected in the progression blood samples of patients harboring a pre-treatment activating *EGFR* mutation and not in either patients or cell lines with wild-type *EGFR*.

## Discussion

Emergence of T790M-driven resistance during EGFR-directed TKI treatment in patients with activating *EGFR* mutations is well described. The presence of T790M in *EGFR* wild-type patients treated with TKI has not been investigated in detail. Traditionally, re-biopsies taken at the time of progression would be needed to study this. However, the opportunity to study tumor DNA in blood samples offers a possibility to overcome this limitation. Here we present data showing that the T790M mutation is not found in the blood of 128 *EGFR* wild-type patients at the time of progression. In comparison, the mutation is present in 38% (6/16) of patients with an activating *EGFR* mutation before treatment.

We further supported our findings by in vitro studies, where we generated *EGFR* wild-type erlotinib-resistant cell lines, none of which acquired the T790M mutation as a resistance mechanism to erlotinib. In contrast, the development of T790M mutations in lung cancer cell lines harboring an activating *EGFR* mutation is well described [13–16].

To our best knowledge, this is the first study to investigate the presence of EGFR T790M as resistance mechanism in a wild-type *EGFR* patient cohort. Prior studies have preferably investigated epithelial-to-mesenchymal transition (EMT) as a mechanism of both acquired and intrinsic resistance mechanism. The majority of these find that EGFR TKI sensitivity both in vitro and in vivo is correlated with an epithelial phenotype [17–20], though conflicting results has also been presented [21]. Furthermore, an in vitro study have shown that activation of insulin-like growth factor 1 receptor may act as mechanism of acquired resistance to EGFR TKI in a wild-type EGFR NSCLC cell line [22]. In the present study, we have not investigated these mechanisms but focused on the observation that the T790 M mutation in EGFR does not appear in an EGFR wild-type background.

## Conclusion

Our results suggest that erlotinib-treated patients with wild-type *EGFR* develop resistance through other mechanisms than T790M, and molecular diagnostics aimed at identifying the responsible events should not focus on T790M.

## Additional file

Additional file 1 **Figure S1.** The erlotinib response of parental and resistant cell lines. A and B: H1568 parental cell line along with H1568 ER and HD. C and D: H1666 parental cell line and H1666 ER and HD. E and F: Calu-3 parental cell line and Calu-3 ER and HD. G and H: H358 parental cell line and H358 ER and HD. ER: erlotinib-resistant cell lines generated by the stepwise escalation method. HD: erlotinib-resistant cell lines generated by the high dose method. (TIFF 1290 kb)

## Abbreviations

cfDNA: cell free DNA
ddPCR: droplet digital PCR
EGFR: Epidermal growth factor receptor
HGF: Hepatocyte growth factor
MET: Met proto-oncogene (hepatocyte growth factor receptor)
NSCLC: Non-small cell lung cancer
RECIST: Response evaluation criteria in solid tumors
