# Supplementary figures and images for: The T790M resistance mutation in EGFR is only found in cfDNA from erlotinib-treated NSCLC patients that harbored an activating EGFR mutation before treatment
Source: BMC Cancer. 2018 Feb 15;18:191. doi: 10.1186/s12885-018-4108-0 (PMC5815238; doi:10.1186/s12885-018-4108-0)

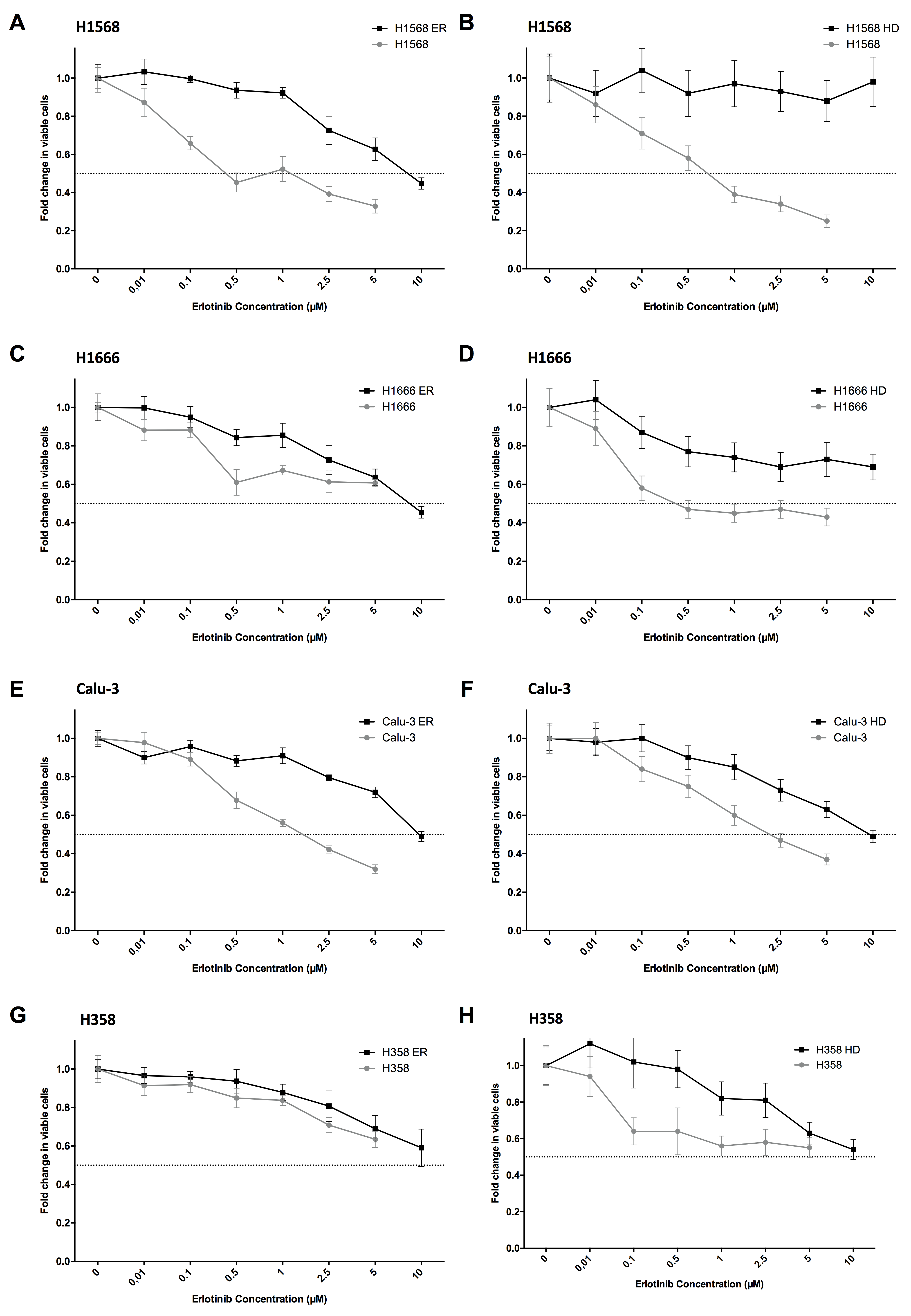

Supplement: Supplementary file 1 — Figure S1. The erlotinib response of parental and resistant cell lines. A and B: H1568 parental cell line along with H1568 ER and HD. C and D: H1666 parental cell line and H1666 ER and HD. E and F: Calu-3 parental cell line and Calu-3 ER and HD. G and H: H358 parental cell line and H358 ER and HD. ER: erlotinib-resistant cell lines generated by the stepwise escalation method. HD: erlotinib-resistant cell lines generated by the high dose method. (TIFF 1290 kb) [file 12885_2018_4108_MOESM1_ESM.tiff]
